# Glycosyltransferases as Markers for Early Tumorigenesis

**DOI:** 10.1155/2015/792672

**Published:** 2015-06-16

**Authors:** Ulrich Andergassen, Friederike Liesche, Alexandra C. Kölbl, Matthias Ilmer, Stefan Hutter, Klaus Friese, Udo Jeschke

**Affiliations:** ^1^Klinik und Poliklinik für Frauenheilkunde und Geburtshilfe, Ludwig-Maximilians-Universitaet Muenchen, Campus Innenstadt, Maistraße 11, 80337 Munich, Germany; ^2^Department of Translational Molecular Pathology, University of Texas MD Anderson Cancer Center, 7435 Fannin Street, Houston, TX 77054, USA

## Abstract

*Background*. Glycosylation is the most frequent posttranslational modification of proteins and lipids influencing inter- and intracellular communication and cell adhesion. Altered glycosylation patterns are characteristically observed in tumour cells. Normal and altered carbohydrate chains are transferred to their acceptor structures via glycosyltransferases. Here, we present the correlation between the presence of three different glycosyltransferases and tumour characteristics. *Methods*. 235 breast cancer tissue samples were stained immunohistochemically for the glycosyltransferases N-acetylgalactosaminyltransferase 6 (GALNT6), *β*-1,6-N-acetylglucosaminyltransferase 2 (GCNT2), and ST6 (*α*-N-acetyl-neuraminyl-2,3-*β*-galactosyl-1,3)-N-acetylgalactosamine *α*-2,6-sialyltransferase 1 (ST6GALNac1). Staining was evaluated by light microscopy and was correlated to different tumour characteristics by statistical analysis. *Results*. We found a statistically significant correlation for the presence of glycosyltransferases and tumour size and grading. Specifically smaller tumours with low grading revealed the highest incidences of glycosyltransferases. Additionally, Her4-expression but not pHer4-expression is correlated with the presence of glycosyltransferases. All other investigated parameters could not uncover any statistically significant reciprocity. *Conclusion*. Here we show, that glycosyltransferases can identify small tumours with well-differentiated cells; hence, glycosylation patterns could be used as a marker for early tumourigenesis. This assumption is supported by the fact that Her4 is also correlated to glycosylation, whereas the activated form of Her4 does not show such a connection with glycosylation.

## 1. Introduction

Glycosylation is the most common posttranslational modification of proteins and lipids, creating structural diversity [1]. The addition of carbohydrate chains influences diverse mechanisms such as cell-cell adhesion [2, 3], communication of cells with their environment [4], or antigen recognition by the immune system [5]. There are two major forms of glycosylation: first, the so-called N-glycosylation. During this process, oligosaccharide precursor chains are covalently linked to Asparagine-residues of proteins. The second form is called O-glycosylation. Here, in a first step, GalNAc residues are attached to Serine- or Threonine-residues under the control of ppGalNac-transferases [6–8]. These carbohydrate residues are later modified in a tissue-specific manner [9, 10].

Cancer cells and tissues are often characterized by an altered glycosylation pattern [11, 12]. As early as 1985, it was shown that cancer tissues stain positive with antibodies against abnormal carbohydrate chains [13]. Many of these tumour associated carbohydrate antigens (TACAs) are well known and described, for example, T-, Tn-, sTn-, and the Lewis-antigens [14, 15], and have been studied extensively in cell culture models [16, 17]. TACAs seem to regulate cellular functions such as signal transduction, antigenicity, interaction with immune effector cells, and cell-cell adhesion [18–22]. Taken together, altered glycosylation seems to contribute to tumourigenesis and tumour progression [23] and hence might offer new targets for diagnosis, prognosis, and therapeutic strategies [24–26].

In breast cancer, altered glycosylation has been linked to a worse prognosis and a shortened overall survival before [27]. The most frequent alterations of glycosylation in breast cancer are shortened O-glycans [28] and increased sialylation [29]. Moreover, altered glycosylation effects the morphological transformation of tumour cells which can ultimately lead to metastasis formation [6].

The main reason for the occurrence of altered glycosylation is a change in the expression of glycosyltransferases—the responsible enzymes for glycosylation [30, 31]. These enzymes are located in the membranes of the endoplasmic reticulum and the Golgi apparatus and transfer carbohydrate chains to acceptor molecules. They are categorized in different subfamilies with regard to the respective transferred carbohydrate. Although the families share almost no sequence homologies and do not have comparable exon-intron-structures, protein domain structures are rather similar: they mostly have an N-terminal cytoplasmatic tail, a signal anchor domain of 16–20 amino acids, an extended stem region, and a catalytic C-terminal domain [32, 33]. Expression profiling of glycosyltransferases has been studied extensively [34–38] and it was reported that oncogenic transformation is regulated at the transcriptional level [39]. Therefore, the expression of glycosyltransferases is an important marker for tumour prognosis and therapeutic outcome [40, 41].

In the present study, we sought to determine correlations between the incidence of glycosyltransferases and other tumour characteristics, such as histology, grading, tumour size, expression of Her2 and Her4, or hormone receptor status. For that purpose, three different glycosyltransferases, namely, N-acetylgalactosaminyltransferase 6 (GALNT6), *β*-1,6-N-acetylglucosaminyltransferase 2 (GCNT2), and ST6(*α*-N-acetyl-neuraminyl-2,3-*β*-galactosyl-1,3)-N-acetylgalactosamine *α*-2,6-sialyltransferase 1 (ST6GALNac1), were immunohistochemically investigated in paraffin-embedded tumour tissue sections. GALNT6 is involved in the first steps of O-glycosylation [42]. It is known that breast cancer expresses GALNT6 mRNA and this phenomenon is mainly associated with smaller tumours (T1) [43]. GCNT2 is related to metastasis formation and influences cell proliferation, migration, and invasion of endothelial cells [44]. ST6GALNac1 synthesizes the sTn-antigen, which is known to be overexpressed in epithelial cancers like breast cancer. Moreover, the expression of ST6GALNac1 results in increased cell migration and reduced cell adhesion [16].

It could be shown that GALNT6 especially is correlated to a small tumour size and low grading, meaning that small, still good differentiated tumours are glycosylated, and thus glycosylation is a marker for a good prognosis for therapy and outcome. Furthermore a correlation of all three glycosyltransferases with Her4, but not with the activated form of Her4, pHer4, could be seen. Her4 is also known to be mostly expressed in tumour tissues which are still more differentiated [45], supporting our hypothesis.

## 2. Materials and Methods

### 2.1. Tumour Tissue Samples

Tumour tissue samples of 235 breast cancer patients undergoing breast cancer surgery between July 1998 and May 2000 were collected (ethical vote 048-08 and 148-12, Ludwig-Maximilians University of Munich, compliant to the Declaration of Helsinki), subsequently embedded in paraffin, and archived. Patient samples in this study were not preselected for certain criteria and therefore show different tumour characteristics with respect to age at time of surgery, histology, grading, tumour size, nodal state, formation of remote metastasis, and hormone receptor state.

**Figure 1:**
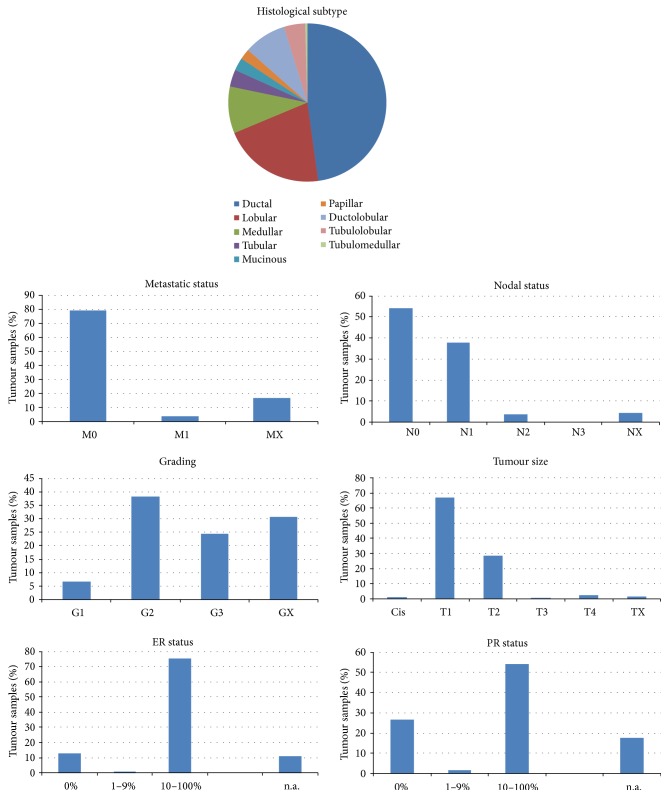
Characteristics of tumour samples used for staining of glycosyltransferases.

Tissue samples can be assigned to characteristics shown in Figure 1.

### 2.2. Immunohistochemistry

The paraffin-embedded samples were cut into 2-3 *μ*m thick sections with a sliding microtome, subsequently placed on specially covered microscope slides (SuperFrost Plus, Menzel GmbH, Braunschweig), and air-dried over night at 56–58°C. For immunohistochemical staining, samples were deparaffinized with xylol (Merck, Darmstadt, Germany) for 20 min and successively washed with different dilutions of ethanol (100%, 90%, and 75%). To prevent unspecific staining of tissue samples, endogenous peroxidase activity was reduced by incubation of the samples in 3% H_2_O_2_ (VWR International, Radnor, USA) for 20 min. Afterwards samples underwent further washes in ethanol and water. Next, antigen retrieval was carried out in boiling Na-citrate buffer (pH 6.00) for 5 min. (Merck, Darmstadt, Germany). After cooling down, tissue samples were washed again in water and PBS (Biochrom, Berlin, Germany).

The prepared slides were first blocked in 10% normal goat serum for 20 min (Vector Laboratories, Burlingame, USA) to prevent unspecific binding of the primary antibody. After removing the blocking solution, primary antibodies were added in optimized concentrations (see Table 1).

Primary antibodies were incubated for 18 h at 4°C. Slides were then washed twice with PBS and subsequently incubated with biotinylated secondary antibodies for 30 min. at room temperature. After removing the secondary antibody, slides were treated with ABC-reagent (Vector Laboratories, Burlingame, USA) according to the manufacturers' instructions for 30 min. Next, DAB-reagent (Dako, Carpinteria, USA) diluted in H_2_O_2_ was added to the slides for 1 min. for ideal staining. Enzyme reaction was stopped by washing the slides in water. Nuclei were counterstained with Hemalaun (AppliChem, Darmstadt, Germany) for 5 min. Last, samples were dehydrated with ethanol and xylol and embedded in Eukitt (Medite, Burgdorf, Germany). The stained samples were then analysed and archived for further evaluations.

**Figure 2:**
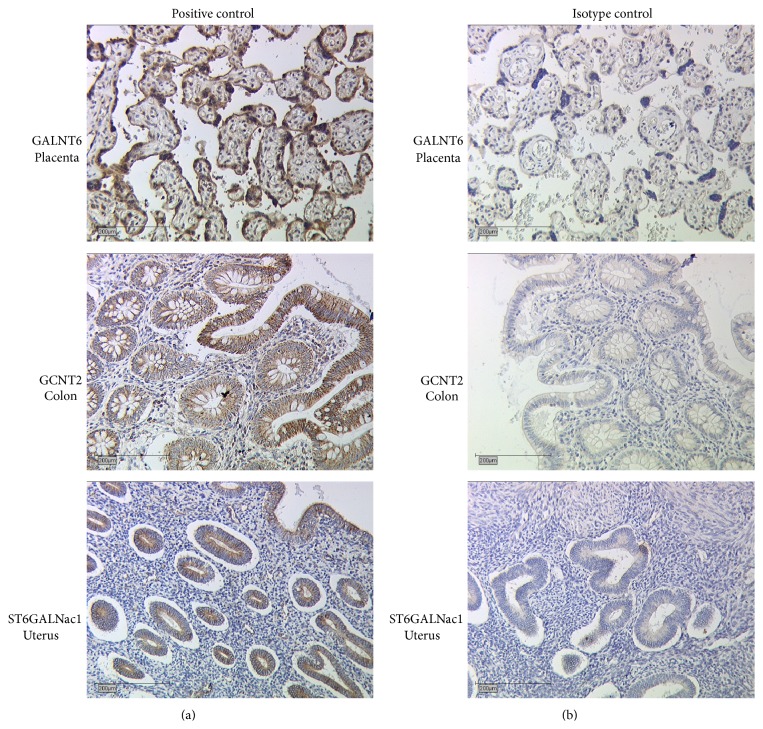
Positive control (a) and isotype control (b). For positive control tissues certainly expressing the questionable antigens were stained. Placental tissue was used for GALNT6 staining, Colon tissue was used for GCNT2 and Uterus was chosen for ST6GALNac1 staining. Furthermore appropriate antibody concentrations were determined in the positive controls. For isotype control same tissues as for positive control are used, but primary antibody is replaced by a control serum, thus excluding unspecific binding signals of the primary antibody.

Before starting the staining procedure on tumour tissue samples positive and isotype control have to be carried out (Figure 2). For positive control a sample from a tissue certainly expressing the antigen of interest is stained to test antibody function and to determine an appropriate dilution of the antibody for staining (Table 1). The isotype control reveals background staining due to primary antibody. Therefore the same tissue used for positive control (GALNT6: Placenta, GCNT2: Colon, ST6GALNac1: Uterus) is stained, but primary antibody is replaced by a control serum.

### 2.3. Microscopy and Evaluation of Staining

**Figure 3:**
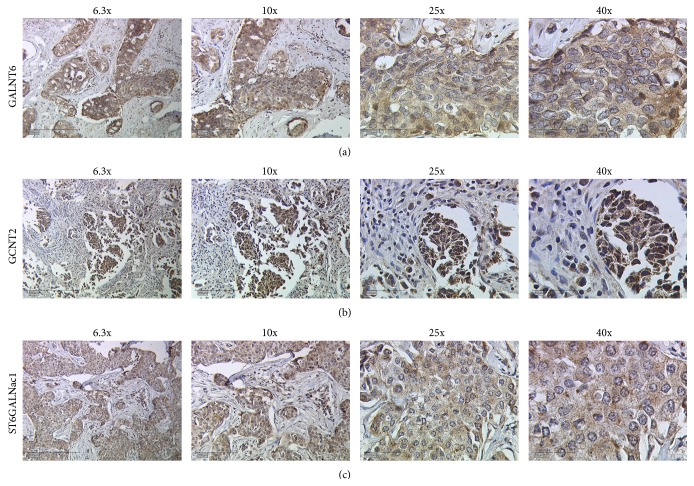
Staining of malignant breast tissue with antibodies against the three glycosyltransferases GALNT6 (a), GCNT2 (b), and ST6GALNac1 (c). Pictures were taken with different objectives (6,3x, 10x, 25x, and 40x; from left to right column) resulting in different magnifications of tissue structures.

Samples were analysed and evaluated with a Leitz Diaplan light microscope (Ernst Leitz GmbH, Wetzlar, Germany). Four objectives with different magnifications (6,3x, 10x, 25x, and 40x) were used (Figure 3; Figure 2: positive and isotype controls).

**Figure 4:**
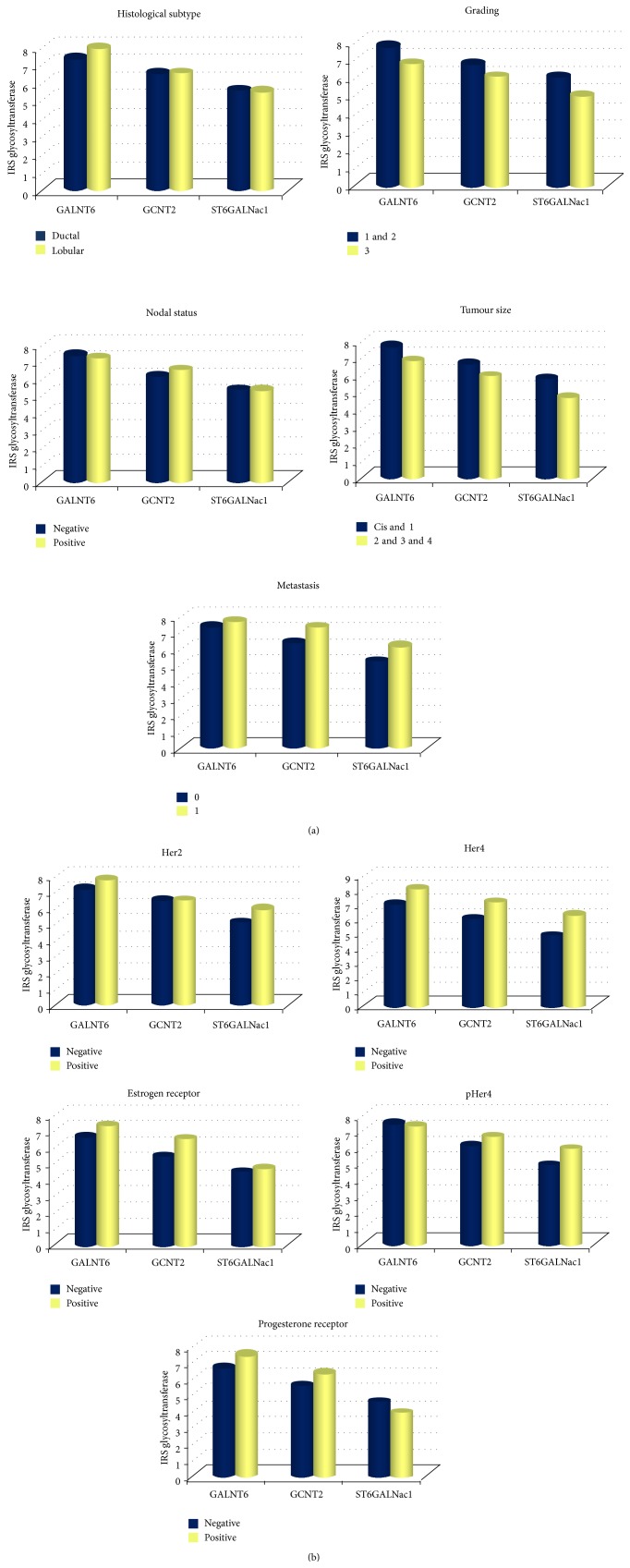
(a) Comparison of IRS-values of glycosyltransferases with histological subtype, nodal status, grading, tumour size, and metastasis. (b) Comparison of IRS-values of glycosyltransferases with Her2, ER, and PR status and Her4 and pHer4.

Stainings were evaluated following the immune-reactive-score (IRS) described by Remmele and Stegner in 1987 [46]. The IRS is obtained by multiplication of staining intensity by the number of stained cells. Staining intensity can be classified into groups from 0 to 3, with 0 being “no staining reaction” and 3 being “strong colour reaction”; numbers of stained cells are classified in a similar manner from 0, “0% stained cells,” to 4 “81–100% stained cells.” Thus, the IRS is in a range from 0 to 12. The IRS of different tumour characteristics were compared (Figure 4).

### 2.4. Statistical Evaluation

**Figure 5:**
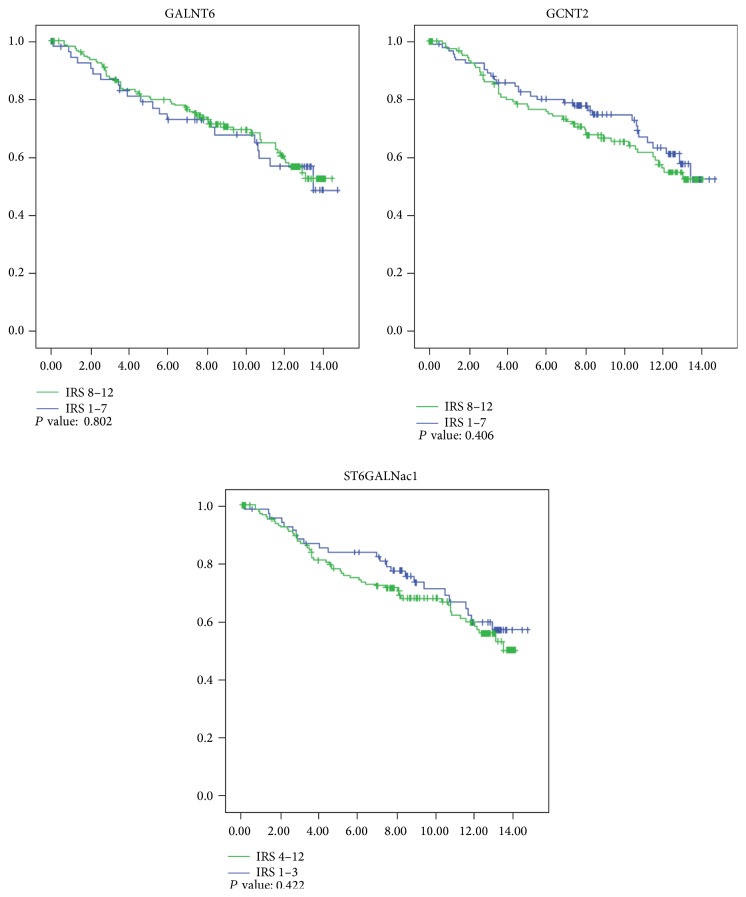
Kaplan-Meier analysis of overall survival with respect to glycosyltransferase expression. *P* values indicate that no differences in overall survival were found in respect to glycosyltransferase expression.

Statistical analysis was done by SPSS (SPSS Inc. Headquarters, Chicago, USA) version 20.0. As patient samples are not normally distributed, nonparametric Mann-Whitney *U* test was applied comparing two variables; for more variables Kruskal-Wallis test was applied. A *P* value of ≤0,05 was regarded as statistically significant (Table 2). Correlations were calculated with the Spearman-Rho test. Survival curves were drawn using Kaplan-Meier analysis (Figure 5).

## 3. Results and Discussion

235 tumour tissue samples were stained for all three different glycosyltransferases (GALNT6, GCNT2, and ST6GALNac1) (Figures 3 and 2 for controls) and their IRS was determined by light microscopic and statistical evaluation. The resulting IRS was then compared to multiple different tumour characteristics in order to detect potential correlations.

First of all, we could not detect any association between the histological subtype and the presence of glycosyltransferases (Figure 4(a)). Lobular and ductal breast cancer revealed similar IRS for all three examined glycosyltransferases with no statistical significant difference (*P* = 0,203, *P* = 0,984, and *P* = 0,904 for GALNT6, GCNT2, and ST6GALNac1, resp.; Table 2). Similarly, we could not detect any significant differences of glycosyltransferase expression in comparison to the nodal status or metastatic setting (Figure 4(a), Table 2). When correlating the tumour grading to the presence of glycosyltransferases, GALNT6 was significantly higher expressed in low grade tumours (grades 1 and 2) compared to high grade tumours (grade 3) (Figure 4(a), Table 2). GCNT2 and ST6GALNac1 were seemingly higher expressed in the same group, however, at a level of borderline significance (*P* = 0,104 for GCNT2 and *P* = 0,094 for ST6GALNac1). With reference to tumour size, we were able to find a similar tendency (Figure 4(a), Table 2). Here, IR-scores for all three enzymes were higher for smaller tumours (Cis and T1) compared to bigger tumours (*T* ≥ 2), with a significant difference for GALNT6 (*P* = 0,012) and borderline significant difference for the other two investigated glycosyltransferases (GCNT2: *P* = 0,066, ST6GALNac1: *P* = 0,059) (Figure 4(a), Table 2). However there seem to be coherences between tumour grading and tumour size and glycosyltransferases. The results suggest that tumours of low grading (grades 1 and 2) are seemingly more dependent on glycosyltransferases than tumours of high grading (grade 3). The latter group contains tumours that are much more dedifferentiated and underwent major changes in their cellular structure making them possibly more independent from glycosyltransferase enzymes. With reference to tumour size, we were able to find a similar tendency (Figure 4(a), Table 2). Furthermore IR-scores for all three enzymes were higher for smaller tumours (Cis and T1) compared to bigger tumours (*T* ≥ 2) leading to the thought that glycosyltransferases are important in early phases of breast tumorigenesis. GALNT6 especially seems to play a role in early tumour formation, a finding that is in consistency with the results of Berois et al. [43]. GALNT6 seems to be characteristic of small, low grade tumours while GCNT2 and ST6GALNac1 are obviously markers of a little more advanced tumour stage, with higher IRS-values in metastatic patient tissue samples and also with a little higher correlation to OAS, which is again in line with former findings [44]. Another correlation was seen between the glycosyltransferases and Her4/pHer4 (Figure 4(b), Table 2), as we detected that the nonphosphorylated form of Her4 seems to correlate strongly with the presence of all three glycosyltransferases (*P* = 0,003, *P* = 0,005, and *P* = 0,001 for GALNT6, GCNT2, and ST6GALNac1, resp.), while the phosphorylated form, pHer4, did not. Only IR-scores of ST6GALNac1 correlated at a statistically significant level with the presence of pHer4 (*P* = 0,039). Her4 is another member of the family of epidermal growth factor receptors and is, hence, a receptor tyrosine kinase. Epidermal growth factor binds to one of the type I transmembrane receptors which leads to a homo- or heterodimerization and subsequently activates the intrinsic kinase domain by autophosphorylation. The phosphorylated domain then serves as starting point for many intracellular signalling cascades [47]. In our observations, we detected that the “inactive,” nonphosphorylated form of Her4 seems to correlate strongly with the presence of all three glycosyltransferases, while the “activated,” phosphorylated form, pHer4, did not correlate at such a strong level. Only IR-scores of ST6GALNac1 correlated at a statistically significant level with the presence of pHer4.

Her2 or the hormone receptors for Estrogen and Progesterone did not reveal any significant correlations to the presence of glycosyltransferases (Figure 4(b), Table 2). Only the presence of Her2 showed a borderline significance with IR-scores for ST6GALNac1 (*P* = 0,077); however, GALNT6 and GCNT2 did not appear to be correlated with Her2.

Last, we tried to evaluate whether expression of glycosyltransferases correlated with the overall survival of patients included into this study. The Kaplan-Meier curves indicate that survival is not dependent on glycosylation (Figure 5), since high or low IR-scores did not deviate significantly (*P* values: GALNT6: 0,802, GCNT2: 0,406, and ST6GALNac1: 0,422).

All these findings are showing and underline a strong coherence between early tumorigenesis and the increased presence of glycosyltransferases.

## 4. Conclusion

From the results presented and discussed in the present study, we conclude that glycosylation of tumour cells does not correlate with tumour histology, formation of metastasis, nodal status, or hormone receptors. On the other hand, glycosyltransferases seem to be abundantly expressed in small and well-differentiated tumours. It is known that altered glycosylation can protect malignant cells from recognition by the immune system [48]. Specifically small and nascent tumours are prone to escape immunosurveillance in order to establish tumour growth and supportive cancer microenvironment. Hence, the overexpression of such enzymes might indicate a useful early step in breast tumorigenesis.

Of the three investigated glycosyltransferases, GALNT6 seems to offer the best correlations with regard to tumour size and grading. Since GALNT6 catalyses early steps in O-glycosylation [43] and therefore plays a central role in the process of glycosylation, it stands out as a potential marker.

A further interesting focus for future research could be to clarify the interrelation between Her4/pHer4 and glycosylation patterns. It is well known that Her4 is heavily glycosylated [45] and could serve in combination with GALNT6 to detect starting and endpoint of the glycosylation cascade. Once Her4 becomes activated by phosphorylation, intracellular signalling pathways are activated, leading to further dedifferentiation of tumour cells. This might eventually lead to a reduction of glycosylation. On the other hand, Her4 is regarded as a marker of favourable prognosis [45], since it is inversely correlated with the histological grading of a tumour [49] and is elevated in sera of early breast cancer patients [50]. In this regard, our data confer the correlation of Her4 to the presence of glycosyltransferases, especially its presence in small tumours and good differentiation.

The above-described hypothesis requires further insights and research. Additionally, the role of ST6GALNac1 should be investigated in more detail, since this glycosyltransferase in particular seems to be correlated with pHer4 and Her2.

It is the task of future research to analyse a wider array of glycosyltransferases for their role in tumour formation and progression, depicting a more detailed scheme of the roles of different glycosyltransferases in early and later tumorigenesis. Additionally the role of glycosyltransferases should be investigated in other gynaecological tumour entities, like ovarian or endometrial adenocarcinomas to gain more detailed insight into the onset of cancer formation.

The results of the presented experiments furthermore give a hint towards the utility of the methodology and the usefulness of glycosyltransferases in terms of tumour characterization. The method is fast and cost-efficient and glycosyltransferases play an important role in tumour development and are independent of processes like epithelial mesenchymal transformation (EMT), so that they could be useful biomarkers in the analysis of tumour tissue samples. This could in turn help to individualize tumour treatment, reducing side effects of any applied therapy while simultaneously increasing the efficiency of a therapy.

## Figures and Tables

**Table 1:** Antibodies used for immunohistochemical staining of breast cancer tissue samples.

Antigen	Host	Isotype	Manufacturer	Positive control tissue	Dilution (in PBS)
GALNT6	Rabbit	Polyclonal IgG	GeneTex	Placenta	1 : 1000
GCNT2	Rabbit	Polyclonal IgG	Novus Biologicals	Colon	1 : 400
ST6GALNac1	Rabbit	Polyclonal IgG	Novus Biologicals	Uterus	1 : 500

**Table 2:** Statistical analysis of the investigated features. Statistically significant *P* values are seen for Her4 (all glycosyltransferases) and for tumour size (GALNT6; GCNT2 and ST6GALNac1 show borderline significance).

	GALNT6 (*P* value)	GCNT2 (*P* value)	ST6GALNac1 (*P* value)
*Histological subtype,* ductal versus lobular	0,203	0,948	0,904
*Nodal status,* negative versus positive	0,532	0,331	0,891
*Metastatic status, * 0 versus 1	0,957	0,383	0,497
*Grading,* 1 and 2 versus 3	0,029	0,104	0,094
*Tumour size,* CIS and 1 versus 2, 3, and 4	0,012	0,066	0,059
*Her4, * negative versus positive	0,003	0,005	0,001
*pHer4, * negative versus positive	0,622	0,113	0,039
*Her2 status, * negative versus positive	0,142	0,925	0,077
*Estrogenreceptor status, *negative versus positive	0,378	0,125	0,672
*Progesterone receptor status,* negative versus positive	0,324	0,266	0,575
